# Efficacy and safety of DMB-I (latrepirdine) therapy in mild to moderate dementia in Alzheimer’s disease: results of a multicenter, double-blind, randomized, placebo-controlled, clinical trial in three parallel groups

**DOI:** 10.1038/s41598-026-49538-4

**Published:** 2026-05-15

**Authors:** Boris I. Gorin, Stanislav O. Pozdnyakov, Ksenia A. Potapova, Elena A. Tukhovskaya, Dmitriy S. Gorchakov

**Affiliations:** 1 Bigespas Ltd, London, UK; 2 Yaroslavl State Medical University, Yaroslavl, Russia; 3 Moscow State University, Moscow, Russia; 4 Shemyakin and Ovchinnikov Institute of Bioorganic Chemistry (Branch), Russian Academy of Sciences, Pushchino, Russia; 5 LLC iPharma, Moscow, Russia

**Keywords:** Alzheimer’s Disease, Dementia, Latrepirdine, Crystalline Polymorphs, Clinical Trial, Diseases, Drug discovery, Health care, Medical research, Neurology, Neuroscience

## Abstract

Alzheimer’s disease (AD) is one of leading dementia causes, affecting over 50 million people worldwide. A multicenter, double-blind, randomized, placebo-controlled clinical trial in three parallel groups was conducted to evaluate efficacy and safety of Latrepirdine polymorph DMB-I in 26-week treatment of dementia in patients with AD. 135 patients of both sexes aged 60 to 90 years were randomized into three groups: DMB-I + placebo, 30 mg/day; DMB-I, 60 mg/day and Placebo. Treatment efficacy was assessed using Alzheimer’s disease Assessment Scale cognitive subscale (ADAS-cog), Mini-Mental State Examination (MMSE-2), quality of life questionnaire (QOL-AD), Clinical Global Impression scale (CGI) and Instrumental Activities of Daily Living score (IADL). Clinical parameters and adverse events (AEs) also assessed. The efficacy of DMB-I at a dose of 60 mg/day was demonstrated by assessing the primary efficacy criterion, namely, a significant change in the ADAS-cog score after 26 weeks of treatment compared with baseline. Severity of cognitive impairment dynamics on ADAS-cog, CGI and LADL scales was significantly improved in groups receiving DMB-I at both doses, compared with placebo. AEs overall incidence was similar between DMB-I and Placebo groups. 26-week therapy with DMB-I at both doses demonstrated efficacy and favorable safety profile. 60 mg/day dose was selected as optimal dose for further studies. Study retrospectively registered on Clinicaltrials.gov on February 27, 2024, NCT06292351.

## Introduction

According to the WHO, 57 million people worldwide were diagnosed with dementia in 2021. By 2030, this number is expected to increase to 82 million, and to 152 million by 2050^1,2^. The most common causes of cognitive impairment are AD and vascular dementia^3^. Sporadic AD is most common and begins after 65–70 years of age. However, in approximately 5–10% of cases, AD has an early onset (hereditary nature), with the onset of symptoms at the age of 40–50 years^4^. The FDA has approved several drugs that are currently standard treatments for dementia, providing symptomatic relief and functional stabilization. Specific drugs used only for the treatment of confirmed Alzheimer’s disease (for example, using PET-CT-Aβ) are aimed at reducing the amount of amyloid plaques in the brain. Anti-amyloid monoclonal antibodies, such as lecanemab and donanemab, are effective in the early stages of the disease and prevent its progression. Drugs used to alleviate the symptoms of the disease, used in later stages of the disease, as well as in unconfirmed (suspected) Alzheimer’s disease, include cholinergic inhibitors donepezil, rivastigmine, and galantamine; and the NMDA glutamate receptor antagonist memantine^5–8^. Among the drugs currently being studied, the most frequently used are GV-971, aducanumab, melatonin, and ginkgo biloba^9^. Latrepirdine is an antihistamine drug, originally developed and used as an antiallergic agent, that was shown to be active in improving cognitive function in AD first in a small clinical trial^10^, and then in a large phase II clinical trial was conducted, in which Latrepirdine was administered to 183 patients for 52 weeks. This study provided compelling evidence for the efficacy of latrepirdine in improving cognitive function in patients with AD^11^. However, a multinational study involving 598 patients with mild to moderate AD, initiated by Medivation and Pfizer, was terminated midway due to lack of effect^12,13^. Despite Pfizer’s disappointing results, research into latrepirdine as a potential treatment for AD continues^14–20^. Preclinical studies show that latrepirdine enhances hippocampal-dependent learning in C57BL6N mice^21^, protects against motor decline and the accumulation of tau-positive degenerative neurons characteristic of the tau^P301S^ transgenic mouse model of AD^22^.Several studies have shown that the neuroprotective effect of latrepirdine is realized through several mechanisms, including reduction of oxidative stress and β-amyloid toxicity, reduction of glutamate neurotoxicity, activation of autophagy and inhibition of mitochondrial permeability pore function^23,24^.

In our previous study, we demonstrated that the crystal structure (polymorphism) directly affects the bioavailability and pharmacological activity of latrepirdine. A new polymorph of latrepirdine, DMB-I^25^, with improved pharmacological properties was discovered. DMB-I demonstrated increased bioavailability in the brain and blood and contributed to the reversal of cognitive impairment in a rat model of scopolamine-induced AD^26^. Regarding the previously noted discrepancies in the results of clinical trials, they can be explained by the fact that none of the previous studies took into account latrepirdine polymorphisms, and therefore the bioavailability of the studied drug in each study could vary. In our clinical study, we used the latrepirdine polymorph DMB-I, which has high bioavailability in the brain and has proven effective in an animal model of AD^25,26^. For compassionate reasons, all patients in our study received background therapy for AD with memantine^27^, as the study was long-term and patients with AD must receive continuous background therapy to avoid rapid deterioration of their condition. Based on the results of clinical trials conducted worldwide, the favorable safety profile of latrepirdine in the treatment of patients with neurodegenerative diseases has been confirmed^28^, which is extremely important since the patients are elderly people with multiple comorbid somatic pathologies^29^. This clinical trial, a phase II study, was conducted to establish the efficacy and optimal dose of a DMB-I in the treatment of dementia in patients with mild to moderate AD. According to current guidelines^30^, the diagnosis of AD can only be established after histological analysis or after analysis in accordance with the ATN criteria; otherwise, the diagnosis is considered probable AD. However, for ease of understanding, we will refer to the patient’s diagnosis as AD in the text.

## Methods

### Study design

**Fig. 1:**
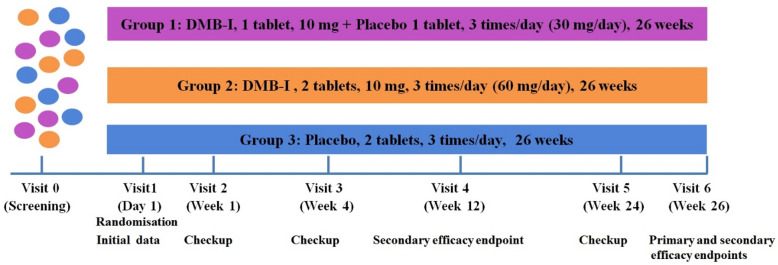
Clinical trial outline. Visit 0 (screening) – Screening duration no more than 14 days. Collection of baseline patient information and assessment of inclusion/exclusion criteria, patient and caregiver signature, patient complaints, demographic and anthropometric data, medical and pharmacotherapeutic history, physical examination, measurement of blood pressure, heart rate, respiratory rate, body temperature, 12-lead ECG, complete blood count, blood chemistry, determination of glomerular filtration rate using the Cockcroft-Gault formula, urinalysis, MRI/CT of head (if not performed within last 12 months), MMSE-2 assessment, and Hamilton scale. Visit 1 (randomization + baseline data). Randomization and first day of therapy into one of three treatment groups. Physical examination, collection of patient complaints, measurement of blood pressure, heart rate, respiratory rate, and body temperature, distribution of a diary and training in its completion, assessment of scales and questionnaires (ADAS-cog, QOL-AD, CGI, IADL). Visit 2, Visit 3 and Visit 5 (examination) – physical examination, collection of patient complaints, measurement of blood pressure, heart rate, respiratory rate, body temperature, assessment of concomitant therapy, assessment of exclusion criteria, assessment and recording of adverse events, verification of patient diary completion. Visit 4 (Checkup + Secondary efficacy endpoint) and Visit 6 (Checkup + Primary efficacy endpoint): physical examination, collection of patient complaints, measurement of blood pressure, heart rate, respiratory rate, body temperature, body weight, 12-lead ECG, complete blood count, blood chemistry, determination of glomerular filtration rate using the Cockcroft-Gault formula, and complete urinalysis, assessment of scales and indices (ADAS-cog, MMSE-2, QOL-AD, CGI, IADL), concomitant therapy, exclusion criteria, adverse events, verification of patient diary completion.

A multicenter, double-blind, randomized, placebo-controlled, dose-titration clinical trial in three parallel groups was conducted to evaluate efficacy and safety of study drug DMB-I in treatment of dementia in AD. Patients diagnosed with mild to moderate dementia in AD received background therapy with memantine at a dose of 20 mg/day. Study involved 135 patients of both sexes aged 60 to 90 years. Patients were randomized into three groups: Group 1: DMB-I 1 tablet, 10 mg + placebo 1 tablet three times a day (daily dose 30 mg); Group 2: DMB-I 2 tablets, 10 mg three times a day (daily dose 60 mg); and Group 3: placebo 2 tablets three times a day. Duration of treatment was 26 weeks. Patients underwent preliminary screening to meet inclusion/exclusion criteria. Study included a total of six patient visits. Efficacy was assessed by primary and secondary efficacy endpoint was assessed after 12 and 26 weeks of treatment. Safety was also assessed based on frequency and severity of AEs, as well as laboratory and physical examination results. Study design is schematically presented in Fig. 1.

### Inclusion criteria

Study included patients who provided informed consent to participate, were aged 60 to 90 years, had an established diagnosis of mild to moderate AD dementia, were receiving basic therapy with memantine at a daily dose of 20 mg for at least 2 months, had a score on MMSE-2 scale 10–23, had no signs of vascular dementia according to CT/MRI data, had a caregiver willing to monitor drug intake and accompany patient to all visits, and were able to undergo tests stipulated by protocol.

### Exclusion criteria

Diagnosis or presence of other diseases causing dementia, other neurodegenerative diseases (Parkinson’s disease, multiple sclerosis, uncontrolled epilepsy, etc.), stroke, active oncological process, need for surgical interventions, disorders of central nervous system and cardiovascular system, disorders of endocrine and digestive systems, taking drugs that negatively affect cognitive functions (tricyclic antidepressants, antipsychotics, etc.), use of prohibited therapy drugs (Cerebrolysin, Ginkgo Biloba extract preparations, any other drugs with nootropic, antioxidant, metabolic action, as well as drugs used to treat dementia), smoking, alcoholism, drug addiction, systemic infections (HIV, etc.), participation in another clinical trial within last 6 months.

### Drugs

Study used test drug DMB-I (Latrepirdine polymorph)^27^ tablets containing 10 mg of active substance, manufactured by Organica JSC, Russia, batch 10,623, best before 06.2026, batch 21,123, best before 11.2026. Placebo tablets that did not contain test substance, was also manufactured by Organica JSC, Russia, batch 10,623, best before 06.2026, batch 21,123, best before 11.2026. Tablets were identical in appearance.

### Test facilities and ethical review

Study was conducted in seven test facilities of Russia: City Hospital No. 40, Saint Petersburg, Russia; LLC “Center For Evidence-Based Medicine”, Yaroslavl, Russia; Sechenov University, Moscow, Russia; Leningrad Regional State Psychoneurological Dispensary, Roshchino, Russia; Federal Center of Brain Research and Neurotechnologies of Federal Medical and Biological Agency (FCBRN of FMBA of Russia), Moscow, Russia; Medical Center Nova Vita, Rostov-on-Don, Russia; State Autonomous Institution of Health Interregional clinical diagnostic center, Kazan, Russia. Ethical review and ethical support of clinical trial were carried out by Local Ethics Committees at all test facilities.

### Declaration of quality assurance for clinical trial

Study was carried out in accordance with requirements of Guidelines on Good Clinical Practice of International Council for Harmonization (ICH GCP) and Eurasian Economic Union (EAEU GCP), principles specified in Declaration of Helsinki and current laws of Russian Federation. All data was entered from primary documentation into electronic Case Report Form (eCRF). The completeness and accuracy of the eCRF were regularly verified by monitors during study facility audits.

### Patient confidentiality declaration

Complete confidentiality of patient’s personal medical information obtained during study was ensured by assigning an identification number, which was used in place of the patient’s last name.

### Data masking – blinding

Double-blinding was maintained throughout study. To ensure blinding and prevent both patients and investigators from becoming unaware of medication being administered, identical coded labeling of medications dispensed to patients and centralized randomization in Interactive Web Response System (IWRS) were used.

### Groups randomization

IWRS integrated into eCRF system was used for randomization of patients to DMB-I or placebo groups in 1:1:1 ratio. Patients were selected by screening (lasting up to 14 days) to collect baseline information and assess inclusion/exclusion criteria. Information on groups and doses is presented in Table 1.

**Table 1:** Change in ADAS-cog score after 26 weeks of treatment compared with baseline (Δ, Visit6 – Visit1).

Parameter	Group 1 (DMB-I + placebo)	Group 2 (DMB-I)	Group 3 (Placebo)
N	46	40	44
MEAN (SD)	-2.52 (5.92)	-3.48 (5.23)	-0.11 (6.72)
SEM	0.87	0.83	1.01
Median; IQR	-1.00; 7.00	-4.00; 6.00	1.00; 6.25
Q1; Q3	-6.00; 1.00	-6.00; 0.00	-2.25; 4.00
Min; Max	-21.00; 8.00	-18.00; 10.00	-21.00; 10.00
Range of variation	29.00	28.00	31.00
Mean difference [Two-sided 97.528% CI for mean difference]	-2.41 [-5.46; 0.65]*	-3.36 [-6.35; -0.37]*	
p-value (one-tailed t-test)**	0.0376	**0.006**	
Mean difference [Two-sided 95% CI for mean difference]	0.95 [-1.44; 3.34]		
p-value (two-tailed t-test)	0.430		

* Results are presented for the respective treatment group compared to the placebo group 3 ** Testing a one-sided superiority hypothesis with a margin of superiority of 0 points for the difference in mean changes in ADAS-cog scores compared to placebo. The hypothesis was tested at a one-sided significance level of α = 0.0125 p-values indicating statistical significance of differences are shown in bold.

### Description of methods for assessing AD severity symptoms

AD Assessment Scale-Cognitive (ADAS-cog) consists of 11 patient-administered tasks to assess cognitive domains of memory, language, and praxis^31^. The higher overall score, the more pronounced cognitive impairment.

Mini-Mental State Examination, 2^ND^ Edition (MMSE-2, www.parinc.com) involves patient completing several tasks to assess cognitive domains such as orientation, immediate memory, attention, arithmetic, and verbal and speech recall. The higher overall score on this test, the less severe cognitive impairment^32^.

QOL-AD questionnaire assesses patients’ quality of life across various aspects, including physical health, energy level, relationships with loved ones, memory, self-esteem, and ability to perform household chores and engage in leisure activities. The higher the overall score, the better patient perceives their quality of life.

Assessment of patient’s condition using Clinical Global Impressions (CGI) scale is performed by physicians based on their clinical experience. CGI scale consists of three subscales: (1) Current severity of patient’s disease - CGI-S (assessed at Visits 1, 4 and 6 on a 7-point scale); (2) Overall improvement in condition - CGI-I (assessed at Visits 4 and 6 on a 7-point scale); (3) CGI Effectiveness Index (a comprehensive scale that assesses both therapeutic efficacy and side effects. Assessed at Visits 4 and 6 on a 16-point scale)^33^. The higher overall score, the worse therapeutic effect.

Instrumental Activities of Daily Living (IADL) scale assesses severity of a patient’s functional impairment in performing daily social activities such as phone calls, shopping, cooking, housekeeping, laundry, medication management, and financial transactions. Total score ranges from 0 (completely dependent on assistance) to 8 (independent)^34^.

### Safety assessment

Safety assessment included an analysis of frequency and severity of treatment-emergent adverse events (TEAEs) recorded from spontaneous reports and complaint collection. Clinically significant changes compared to baseline were assessed for body weight, laboratory test results (clinical and biochemical blood tests, urinalysis), instrumental examination results, physical examination results, vital signs, and 12-lead ECG were assessed. Concomitant therapy initiated during the study and concomitant procedures prescribed during the study were taken into account.

### Efficacy assessment

#### Primary efficacy endpoint

Primary endpoint is difference in mean ADAS-cog score change after 26 weeks of treatment (Visit 6) relative to baseline values obtained from patients at Visit 1. A two-stage comparison was used to select optimal dosage: first, each DMB-I group is compared with placebo, after which DMB-I groups are compared with each other to establish dose-response.

#### Secondary efficacy endpoint

Treatment efficacy was assessed based on change in all studied parameters (ADAS-cog, MMSE-2, QOL-AD, CGI, and IADL) after 12 weeks of therapy (Visit 4) and after 26 weeks of therapy (Visit 6) relative to baseline (Visit 1), as well as differences in these changes between DBM-I and placebo groups. Differences between each DBM-I dose and placebo were first assessed. If such differences were detected, DBM-I doses were compared to determine optimal dose.

### Statistical analysis

Data analysis was performed using specialized software “R Project for Statistical Computing” version 4.0.4^35^. Before beginning study, a power assessment and sample size determination were performed with PASS 2021 software^36^. Group-sequential superiority by margin tests for two means with known variance (Simulation), was used for sample size calculations. O’Brien-Fleming Analog function was used to control for type I error, taking into account interim analyses. Based on a similar study conducted earlier^15^ for this study, it was assumed that difference between DMB-I group at each dose and placebo group at week 26 would be -5.7 points. Sample size calculation was performed to ensure a power of at least 80% with a standard deviation (SD) = 4.4 points, a superiority margin (δ) = 0, a type I error probability α/4 = 0.0125 (taking into account correction for multiple comparisons, corresponding to upper limit of two-sided 97.5% CI less than δ), and subject ratio in groups of 1:1. Ultimately, to achieve a power level of 80% for each group, a sample size of 45 subjects was determined, taking into account a 10% margin in case of early patient dropout.

For efficacy analysis, a statistical model of “superiority” was used for DMB-I compared to placebo. Both groups receiving of DMB-I in different doses were compared separately with placebo group.

For superiority study, null (H_01_) and alternative (H_A1_) hypotheses were formulated as follows:

H_01_: µE – µP ≥ δ, H_A1_: µE – µP < δ, where.

µE and µP are mean changes in ADAS-cog scores at 26 weeks from baseline in treatment groups for each dose of DMB-I (µE) and placebo (µP); δ is margin of superiority of DMB-I treatment compared to placebo. In this study, δ = 0 points was assumed.

Type I error correction was applied, and H_01_ was tested at a significance level of α = 0.025. The efficacy of treatment for DMB-I each dose was considered superior to that of placebo if upper limit of two-sided 97.5% confidence interval for difference in µE and µP values was less than margin of superiority (δ).

If H_01_ was rejected for at least one DMB-I dose, statistical hypothesis of “equality” was tested for two DMB-I dosage groups. For statistical analysis of “equality” hypothesis, we used second-order null hypothesis H_02_, which stated that efficacy of two different drug doses is equal, and alternative hypothesis H_02_, which stated that efficacy of two DMB-I doses differs:

H_02_: µA – µB = 0, H_A2_: µA – µB ≠ 0,

where µA and µB – mean changes in ADAS-cog scores after 26 weeks relative to baseline in drug treatment groups at different dosages.

Since null hypothesis is tested only if hypothesis H_01_ is rejected for at least one of dosages of DMB-I, then, in accordance with testing hierarchy principle, no correction for type I error is required. This hypothesis is tested at the significance level α = 0.05.

Before performing analysis of intergroup differences, normality of distribution was assessed using Shapiro-Wilk test. To analyze significance of differences in parameter changes from baseline value, Mann-Whitney U-test (T/U-test) was used. Between-group differences were analyzed after testing normality of distribution using ANOVA with post-hoc Tukey and Dunnett tests for normal distributions and Kruskal-Wallis for non-normal distributions.

## Results

### Screening and group randomization

**Fig. 2:**
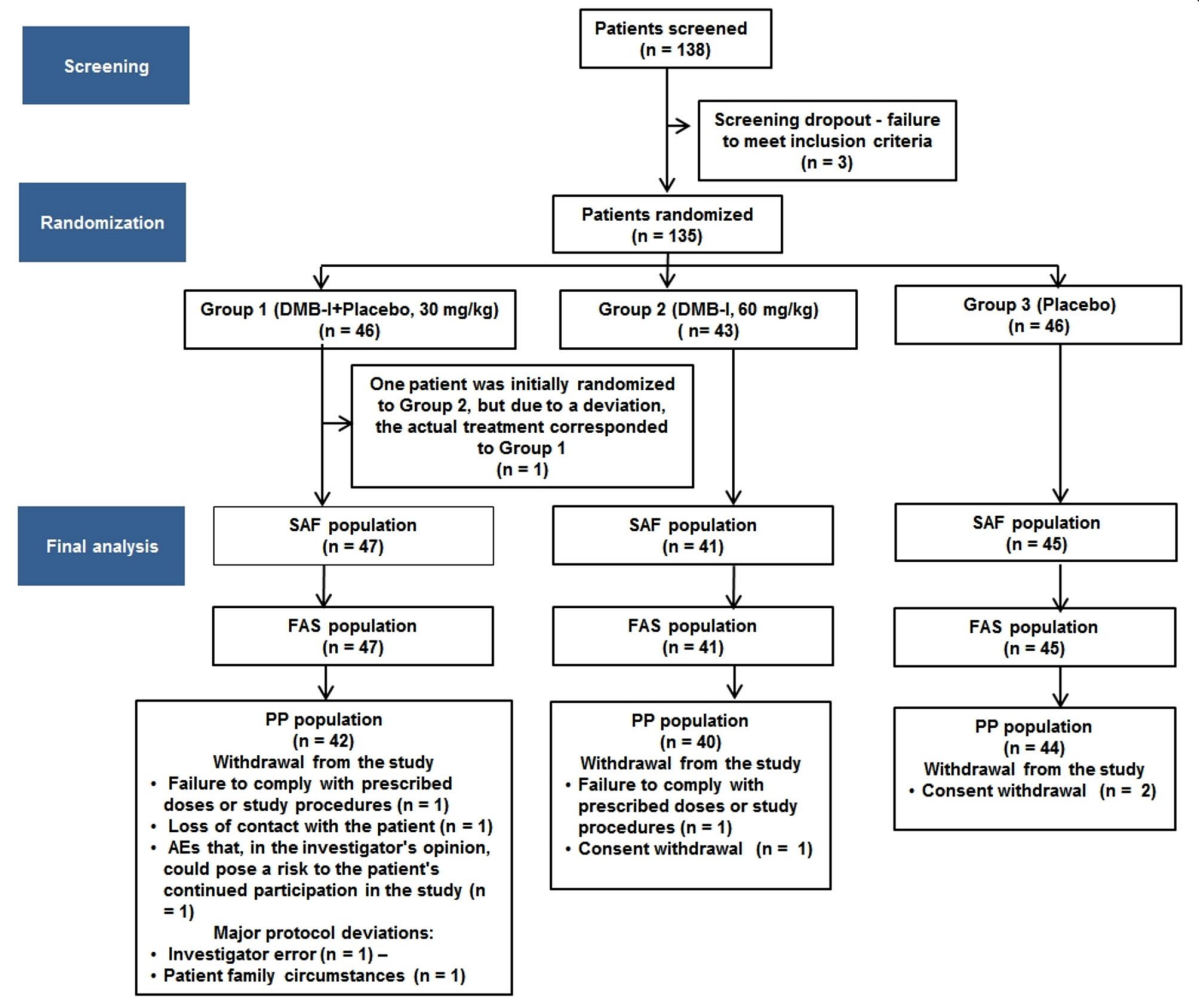
Patient disposition scheme in the study.

A total of 138 patients were screened. A total of 135 patients were randomized in study: 46 patients in Group 1 (DMB-I + placebo), 43 patients in Group 2 (DMB-I), and 46 patients in Group 3 (placebo). (Fig. 2).

The mean age of patients included in analysis was 74 years, with a minimum of 60 years and a maximum of 86 years. Thirty-eight (29%) patients were male and 95 (71%) were female. The mean body weight was 70 ± 12 kg (minimum 46 kg, maximum 110 kg), mean mass index (BMI) was 26 ± 4 kg/m^2^ (minimum 19 kg/m^2^, maximum 36 kg/m^2^).

Thus, patients in all three study groups corresponded to epidemiological data for AD (most patients were older, with a predominance of females). Moreover, all study groups also included patients with early-onset AD (under 65 years old).

### Сoncomitant treatment and comorbidities

It should be noted that all patients randomized at the seven study centers were patients of the physicians conducting this clinical trial, with whom they had established long-term and positive relationships. This allowed for more targeted patient selection, ensured comfortable physician-patient interactions, and reduced the likelihood of loss of contact with the patient during the study. A detailed medical history was collected from all patients before study inclusion. All patients underwent an MRI or CT scan of the brain no earlier than 4.5 months before the start of the study. The patients had the following comorbidities: 78% had vascular disease, with hypertension and atherosclerosis predominating. All patients were menopausal. 44% had metabolic disorders, with dyslipidemia, obesity, and type 2 diabetes mellitus predominating. 41% had heart disease, with myocardial ischemia and chronic heart failure predominating. 35% had musculoskeletal and connective tissue disorders, with osteoarthritis being the predominant type. 32% had nervous system disorders, with cerebral artery sclerosis being the predominant type. Less than 20% of patients had concomitant gastrointestinal and endocrine disorders. Less than 10% had visual and hearing impairments, or liver and kidney disease. 100% of patients included in the study received concomitant medications. Specifically, 100% of patients had taken memantine for at least two months prior to the study. Additionally, 52% of patients were taking drugs that affect angiotensin-converting enzyme, 41% were taking anticoagulants, 23% were taking lipid-lowering drugs, 22% were taking beta-blockers, 17% were taking calcium channel blockers, and less than 10% were taking diuretics, thyroid hormones, vitamins, and mineral supplements.

### Primary efficacy endpoint analysis

The point estimate of difference in mean change between Group 1 (DMB-I, 30 mg/day) and Group 3 (Placebo) was − 2.52 points, with a confidence interval of -5.46 to 0.65 points (one-sided P-value = 0.0376). For Group 1 (DMB-I, 30 mg/day), H_01_ of no efficacy could not be rejected, as confidence interval contains 0, and p-value exceeds established significance level (α = 0.01236). This indicates that no statistically significant difference was found between Group 1 (DMB-I, 30 mg/day) and Group 3 (placebo). The point estimate of difference in mean changes between Group 2 (DMB-I, 60 mg/day) and Group 3 (Placebo) was − 3.36 points, with a confidence interval of -6.35 to -0.37 points (p-value for a one-sided test = 0.006). For this group, H_01_ of lack of efficacy was rejected, as confidence interval did not contain 0, and p-value did not exceed established significance level (α = 0.0125). Therefore, it can be concluded that there is statistically significant evidence of efficacy of Group 2 (DMB-I, 60 mg day) compared to Group 3 (Placebo).

**Fig. 3:**
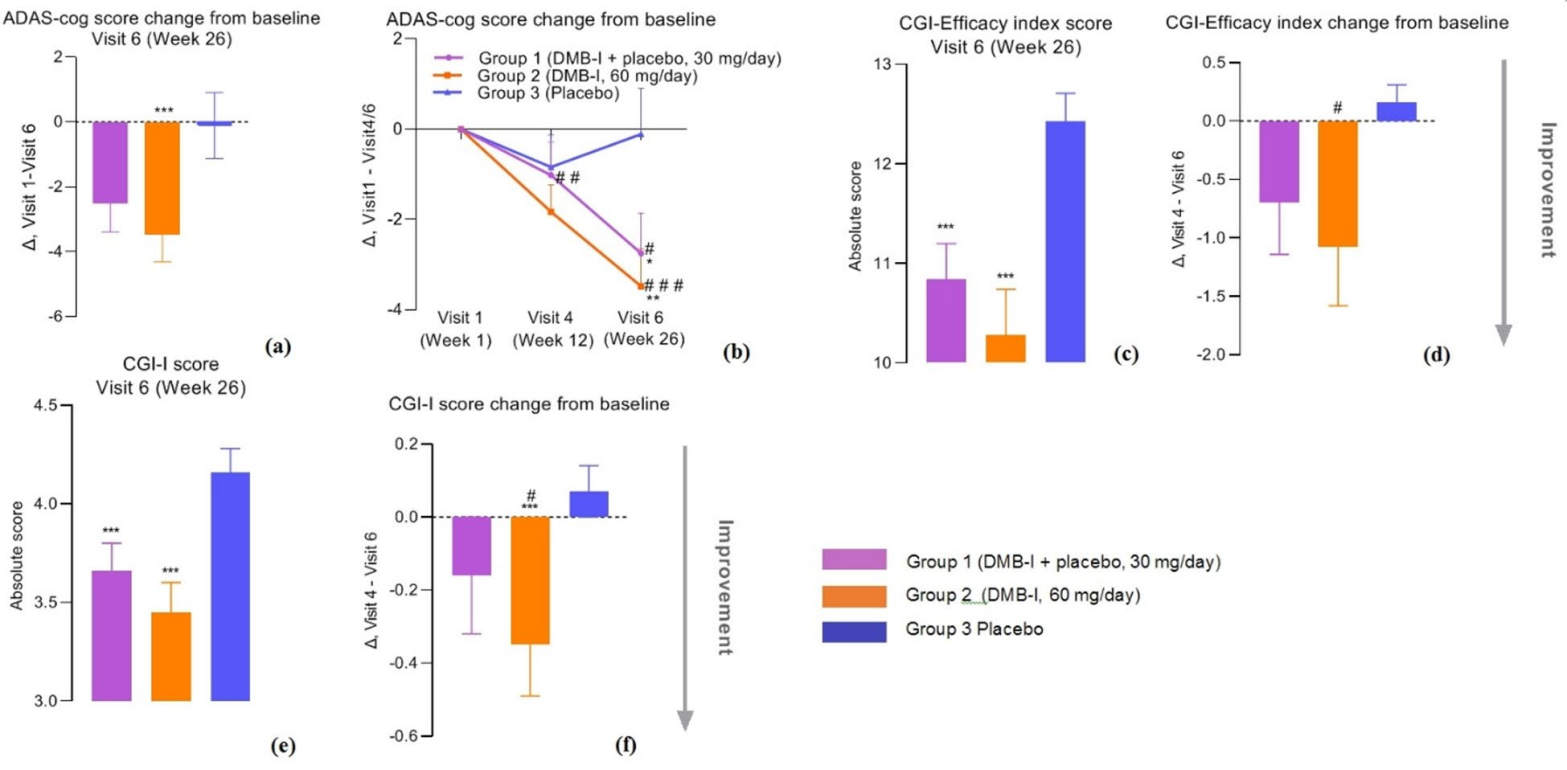
Results of efficacy assessment. (**a**) - primary endpoint of treatment efficacy - mean change - difference between baseline ADAS-cog total score at Visit 1 and ADAS-cog total score after 26 weeks of treatment at Visit 6 (Visit 1 – Visit 6); (**b**) - dynamics of change in ADAS-cog score (Δ - difference between total ADAS-cog score at Visit 4 or Visit 6 and total ADAS-cog score at Visit 1); (**c**) - assessment of overall improvement in patients’ condition according to CGI-Efficacy index absolute score at Visit 6 (Week 26); (**d**) - change in CGI-Efficacy index from baseline, calculated as difference in scores between Visit 4 and Visit 6; (**e**) – CGI-I absolute score at Visit 6; (**f**) - change in CGI-I from baseline, calculated as difference in scores between Visit 4 and Visit 6. Data are presented as MEAN + SEM. @@@ *p* ≤ 0.0125 according to t-test relative to Group 3 (Placebo); #*p* ≤ 0.05, ##*p* ≤ 0.01, ###*p* ≤ 0.001 relative to baseline value at Visit 1 according to T/U-tests for pairwise comparison;, *p ≤ 0.05, **p ≤ 0.01,***p ≤ 0.001 relative to Placebo group according to ANOVA, post-hoc Tukey/Dunnett tests.

The point estimate of difference in mean changes between Group 1 (DMB-I, 30 mg/day) and Group 2 (DMB-I, 60 mg/day) was 0.95 points, with a 95% confidence interval of -1.44 to 3.34 points. Since confidence interval includes 0, this indicates no statistically significant difference between groups (p-value = 0.430). The results do not allow for definitive confirmation of a dose-dependent effect, as difference between groups did not reach statistical significance. Based on analysis, it can be concluded that Group 2 (DMB-I, 60 mg/day) demonstrated statistically significant efficacy compared to Group 3 (Placebo). Results are presented in Table 1; Fig. 3.

### Secondary efficacy endpoint analysis

#### Dynamics of changes in ADAS-cog scale after 12 weeks of therapy (Visit 4) and after 26 weeks of therapy (Visit 6) compared with baseline (Visit 1)

When comparing absolute values of ADAS-cog score between groups after 12 weeks of therapy, no statistically significant differences were found.

When analyzing change from baseline values (Visit 1) after 12 weeks of therapy (Visit 4), statistically significant reduction in ADAS-cog score was observed within Group 2 (DMB-I, 60 mg/day) with a p-value of 0.0037, which indicate positive dynamics already at beginning of treatment (within first 3 months of therapy).

When analyzing changes in ADAS-cog score from baseline (Visit 1) to 26 weeks of therapy (Visit 6), a statistically significant reduction was demonstrated in Group 1 (DMB-I, 30 mg/day + placebo) (p-value change = 0.0036) and in Group 2 (DMB-I, 60 mg/day) (p-value change = 0.0002).

Analysis of between-group differences in ADAS-cog after 26 weeks showed significant differences between Group 1 (DMB-I, 30 mg/day) and Group 3 (Placebo) for absolute scores (p-value = 0.0428). Between-group differences in change from baseline were demonstrated between Group 1 (DMB-I + placebo) and Group 3 (Placebo) (p-value = 0.0410) and between Group 2 (DMB-I) and Group 3 (Placebo) (p-value = 0.0063).

Thus, after 26 weeks of therapy, positive dynamics in improving cognitive function were demonstrated in groups receiving DMB-I at doses of 30 mg/day and 60 mg/day. Data are presented in Table 2; Fig. 3.

**Table 2:** Severity of cognitive impairment dynamics according to ADAS-cog scale. FAS population.

Parameter Visit	Average score			Changes from baseline (Visit 1)		
	Group 1 (DMB-I + placebo)	Group 2 (DMB-I)	Group 3 (Placebo)	Group 1 (DMB-I + placebo)	Group 2 (DMB-I)	Group 3 (Placebo)
Visit 1						
N	47	42	46			
MEAN (SD)	33.98 (9.87)	35.60 (9.38)	35.65 (9.21)			
SEM	1.44	1.45	1.36			
Median; IQR	32.00; 11.00	36.00; 12.00	34.00; 12.75			
Q1; Q3	29.00; 40.00	30.00; 42.00	29.00; 41.75			
Min; Max	14.00; 54.00	13.00; 54.00	18.00; 54.00			
Range of variation	40.00	41.00	36.00			
p-value between groups (ANOVA/Kruskal-Wallis)	0.6320			-		
**Visit 4**						
N	46	40	44	46	40	44
MEAN (SD)	32.70 (8.58)	33.25 (9.49)	34.93 (8.63)	-1.02 (5.04)	-1.83 (3.73)	-0.84 (4.71)
SEM	1.26	1.50	1.30	0.74	0.59	0.71
Median; IQR	32.00; 9.00	33.50; 14.25	34.00; 12.00	-1.00; 5.00	-1.50; 4.00	0.00; 4.50
Q1; Q3	28.00; 37.00	25.75; 40.00	28.00; 40.00	-3.00; 2.00	-4.00; 0.00	-2.25; 2.25
Min; Max	14.00; 53.00	13.00; 50.00	22.00; 53.00	-17.00; 9.00	-12.00; 4.00	-17.00; 8.00
Range of variation	39.00	37.00	31.00	26.00	16.00	25.00
p-value of change (paired T/U-test)				0.1760	**0.0037**	0.4660
p-value between groups (ANOVA/Kruskal-Wallis)	0.4680			0.3420		
**Visit 6**						
N	44	40	44	44	40	44
MEAN (SD)	30.95 (8.81)	31.60 (8.81)	35.66 (9.53)	-2.75 (5.93)	-3.48 (5.23)	-0.11 (6.72)
SEM	1.33	1.39	1.44	0.89	0.83	1.01
Median; IQR	31.00; 11.50	32.50; 11.25	35.50; 11.25	-1.50; 7.25	-4.00; 6.00	1.00; 6.25
Q1; Q3	25.00; 36.50	26.75; 38.00	29.75; 41.00	-6.25; 1.00	-6.00; 0.00	-2.25; 4.00
Min; Max	12.00; 51.00	8.00; 46.00	14.00; 55.00	-21.00; 8.00	-18.00; 10.00	-21.00; 10.00
Range of variation	39.00	38.00	41.00	29.00	28.00	31.00
p-value of change (paired T/U-test)				**0.0036**	**0.0002**	0.5740
p-value between groups (ANOVA/Kruskal-Wallis)	**0.0350**			**0.0064**		
p-value for pairwise comparisons (Tukey/Dunnett)	Group 1 (DMB-I + placebo) - Group 2 (DMB-I) = 0.9430 Group 1 (DMB-I + placebo) - Group 3 (Placebo) = **0.0428** Group 2 (DMB-I) - Group 3 (Placebo) = 0.1050			Group 1 (DMB-I + placebo) - Group 2 (DMB-I) = 0.3568 Group 1 (DMB-I + placebo) - Group 3 (Placebo) = **0.0410** Group 2 (DMB-I) - Group 3 (Placebo) = **0.0063**		

p-values indicating statistical significance of differences are shown in bold.

#### Dynamics of changes in mini-mental state examination (MMSE-2 ) score after 12 weeks of treatment (Visit 4) and after 26 weeks of treatment (Visit 6) compared with baseline (Visit 0)

The conducted between-group analysis revealed no statistically significant differences between groups for absolute values of the MMSE-2 score at Screening, Visits 4 and 6, or for the change in score compared with baseline (Visit 0). Data are shown in Table 3.

**Table 3:** Dynamics of results of MMSE-2 score and indices assessment. FAS population.

Parameter/ Visit	Average score			Changes from Visit 0		
	Group 1 (DMB-I + placebo)	Group 2 (DMB-I)	Group 3 (Placebo)	Group 1 (DMB-I + placebo)	Group 2 (DMB-I)	Group 3 (Placebo)
Visit 0(Screening)						
N	47	42	46			
MEAN (SD)	18.91 (2.79)	18.71 (2.87)	18.78 (2.91)			
SEM	0.41	0.44	0.43			
Median; IQR	19.00; 4.00	19.00; 4.00	19.00; 4.75			
Q1; Q3	17.00; 21.00	17.00; 21.00	16.25; 21.00			
Min; Max	12:00; 23:00	12:00; 23:00	12:00; 23:00			
Range of variation	11:00	11:00	11:00			
p-value between groups (ANOVA/Kruskal-Wallis)	0.9510					
**Visit 4**						
N	46	40	44	46	40	44
MEAN (SD)	19.09 (3.05)	19.25 (3.20)	18.95 (3.08)	0.09 (2.14)	0.40 (2.13)	0.18 (2.00)
SEM	0.45	0.51	0.46	0.32	0.34	0.30
Median; IQR	19.50; 4.00	19.00; 4.00	19.00; 4.00	0.00; 2.00	0.50; 2.25	0.00; 2.00
Q1; Q3	17.00; 21.00	17.00; 21.00	17.00; 21.00	-1.00; 1.00	-1.00; 1.25	-1.00; 1.00
Min; Max	12:00; 26:00	11:00; 27:00	12:00; 25:00	-7.00; 6.00	-5.00; 6.00	-5.00; 5.00
Range of variation	2:00 PM	4:00 PM	13.00	13.00	11:00	10:00
p-value of change (paired T/U-test)				0.9050	0.2430	0.5500
p-value between groups (ANOVA/Kruskal-Wallis)	0.9100			0.5720		
**Visit 6**						
N	44	40	44	44	40	44
MEAN (SD)	19.16 (3.06)	19.48 (3.50)	18.36 (3.13)	0.02 (2.28)	0.63 (2.78)	-0.41 (2.07)
SEM	0.46	0.55	0.47	0.34	0.44	0.31
Median; IQR	19.00; 3.25	20.00; 4.00	18.00; 5.00	0.00; 2.50	0.00; 2.25	0.00; 2.25
Q1; Q3	17.75; 21.00	17.00; 21.00	16.00; 21.00	-1.25; 1.25	-0.25; 2.00	-2.00; 0.25
Min; Max	10:00; 26:00	12:00; 28:00	12:00; 25:00	-6.00; 5.00	-6.00; 7.00	-5.00; 5.00
Range of variation	4:00 PM	4:00 PM	13.00	11:00	13.00	10:00
p-value of change (paired T/U-test)				0.9480	0.1630	0.1970
p-value between groups (ANOVA/Kruskal-Wallis)	0.2650			0.1420		

#### Dynamics of changes in clinical global impression by CGI-Score, CGI-Efficacy index and CGI-Improvement after 12 weeks of therapy (Visit 4) and after 26 weeks of therapy (Visit 6) compared with baseline (Visit 1)

After 26 weeks of therapy, absolute value of total CGI-Efficacy index score was reduced in both groups receiving DMB-I at doses of 30 mg/day and 60 mg/day relative to placebo group (*p* = 0.0016 and *p* = 0.0006, respectively), and change in CGI-Efficacy was significantly reduced in Group 2 (DMB-I, 60 mg/day) relative to its own baseline value (*p* = 0.0425), which together indicates effectiveness of DMB-I therapy.

When assessing overall improvement after 26 weeks of therapy, a significant decrease in absolute CGI-I score was observed in both groups receiving DMB-I at doses of 30 mg/ day and 60 mg/day relative to placebo group (*p* = 0.0045 and *p* = 0.0005, respectively). After 26 weeks of therapy, change in CGI-I in Group 2, receiving DMB-I at a dose of 60 mg/day, was statistically significant both relative to its own baseline (*p* = 0.0235) and relative to placebo group (*p* = 0.0468), indicating a positive effect of DMB-I on overall condition of patients. Data are presented in Table 4; Fig. 3.

**Table 4:** Dynamics of results of CGI Scale and indices assessment. FAS population.

Parameter/ Visit	Average score			Changes from Visit 1		
	Group 1 (DMB-I + placebo)	Group 2 (DMB-I)	Group 3 (Placebo)	Group 1 (DMB-I + placebo)	Group 2 (DMB-I)	Group 3 (Placebo)
CGI-S (The severity of the patient’s disorder at present)						
**Visit 1**						
N	47	41	45			
MEAN (SD)	3.74 (0.64)	3.80 (0.68)	3.78 (0.74)			
SEM	0.09	0.11	0.11			
Median; IQR	4.00; 1.00	4.00; 1.00	4.00; 1.00			
Q1; Q3	3.00; 4.00	3.00; 4.00	3.00; 4.00			
Min; Max	3.00; 5.00	3.00; 5.00	3.00; 6.00			
Range of variation	2.00	2.00	3.00			
p-value between groups (ANOVA/Kruskal-Wallis)	0.9190					
**Visit 4**						
N	46	40	44	46	40	44
MEAN (SD)	3.76 (0.67)	3.80 (0.65)	3.77 (0.71)	0.04 (0.29)	0.03 (0.16)	0.00 (0.30)
SEM	0.10	0.10	0.11	0.04	0.03	0.05
Median; IQR	4.00; 1.00	4.00; 1.00	4.00; 1.00	0.00; 0.00	0.00; 0.00	0.00; 0.00
Q1; Q3	3.00; 4.00	3.00; 4.00	3.00; 4.00	0.00; 0.00	0.00; 0.00	0.00; 0.00
Min; Max	3.00; 5.00	3.00; 5.00	3.00; 6.00	-1.00; 1.00	0.00; 1.00	-1.00; 1.00
Range of variation	2.00	2.00	3.00	2.00	1.00	2.00
p-value of change (paired T/U-test)				0.4240	0.999	
p-value between groups (ANOVA/Kruskal-Wallis)	0.9330			0.7370		
**Visit 6**						
N	44	40	44	44	40	44
MEAN (SD)	3.80 (0.67)	3.83 (0.68)	3.86 (0.67)	0.09 (0.36)	0.05 (0.32)	0.09 (0.42)
SEM	0.10	0.11	0.10	0.05	0.05	0.06
Median; IQR	4.00; 1.00	4.00; 1.00	4.00; 1.00	0.00; 0.00	0.00; 0.00	0.00; 0.00
Q1; Q3	3.00; 4.00	3.00; 4.00	3.00; 4.00	0.00; 0.00	0.00; 0.00	0.00; 0.00
Min; Max	3.00; 5.00	3.00; 5.00	3.00; 6.00	-1.00; 1.00	-1.00; 1.00	-1.00; 1.00
Range of variation	2.00	2.00	3.00	2.00	2.00	2.00
p-value of change (paired T/U-test)				0.1290	0.4240	0.1820
p-value between groups (ANOVA/Kruskal-Wallis)	0.9140			0.8330		
**CGI-Efficacy Index**						
**Visit 4**						
N	46	40	44			
MEAN (SD)	11.65 (2.43)	11.35 (2.32)	12.27 (1.98)			
SEM	0.36	0.37	0.30			
Median; IQR	13.00; 3.00	13.00; 4.00	13.00; 0.00			
Q1; Q3	10.00; 13.00	9.00; 13.00	13.00; 13.00			
Min; Max	5.00; 14.00	5.00; 14.00	5.00; 14.00			
Range of variation	9.00	9.00	9.00			
p-value between groups (ANOVA/Kruskal-Wallis)	0.0753					
**Visit 6**						
N	44	40	44	44	40	44
MEAN (SD)	10.84 (2.36)	10.28 (2.89)	12.43 (1.84)	-0.70 (2.91)	-1.08 (3.16)	0.16 (0.99)
SEM	0.36	0.46	0.28	0.44	0.50	0.15
Median; IQR	10.00; 4.00	9.50; 4.00	13.00; 0.00	0.00; 4.00	0.00; 4.00	0.00; 0.00
Q1; Q3	9.00; 13.00	9.00; 13.00	13.00; 13.00	-4.00; 0.00	-4.00; 0.00	0.00; 0.00
Min; Max	5.00; 14.00	5.00; 14.00	5.00; 14.00	-8.00; 5.00	-8.00; 4.00	-1.00; 4.00
Range of variation	9.00	9.00	9.00	13.00	12.00	5.00
p-value of change (paired T/U-test)				0.1430	**0.0425**	0.4990
p-value between groups (ANOVA/Kruskal-Wallis)	**0.0002**			0.1520		
p-value for pairwise comparisons (Tukey/Dunnett)	Group 1 (DMB-I + Placebo) - Group 2 (DMB-I) = 0.5839 Group 1 (DMB-I + Placebo) - Group 3 (Placebo) = **0.0016** Group 2 (DMB-I) - Group 3 (Placebo) = **0.0006**					
**CGI-I** (**General improvement of condition)**						
**Visit 4**						
N	46	40	44			
MEAN (SD)	3.83 (0.68)	3.80 (0.79)	4.09 (0.74)			
SEM	0.10	0.13	0.11			
Median; IQR	4.00; 1.00	4.00; 1.00	4.00; 0.25			
Q1; Q3	3.00; 4.00	3.00; 4.00	4.00; 4.25			
Min; Max	2.00; 5.00	2.00; 6.00	2.00; 6.00			
Range of variation	3.00	4.00	4.00			
p-value between groups (ANOVA/Kruskal-Wallis)	0.0971					
**Visit 6**						
N	44	40	44	44	40	44
MEAN (SD)	3.66 (0.94)	3.45 (0.93)	4.16 (0.78)	-0.16 (1.03)	-0.35 (0.89)	0.07 (0.45)
SEM	0.14	0.15	0.12	0.16	0.14	0.07
Median; IQR	3.00; 1.00	3.00; 1.00	4.00; 1.00	0.00; 1.00	0.00; 1.00	0.00; 0.00
Q1; Q3	3.00; 4.00	3.00; 4.00	4.00; 5.00	-1.00; 0.00	-1.00; 0.00	0.00; 0.00
Min; Max	2.00; 6.00	2.00; 6.00	2.00; 6.00	-2.00; 2.00	-2.00; 2.00	-1.00; 1.00
Range of variation	4.00	4.00	4.00	4.00	4.00	2.00
p-value of change (paired T/U-test)				0.3360	**0.0235**	0.3510
p-value between groups (ANOVA/Kruskal-Wallis)	**0.0003**			**0.0476**		
p-value for pairwise comparisons (Tukey/ Dunnett)	Group 1 (DMB-I + Placebo) - Group 2 (DMB-I) = 0.3726 Group 1 (DMB-I + Placebo) - Group 3 (Placebo) = **0.0045** Group 2 (DMB-I) - Group 3 (Placebo) = **0.0005**			Group 1 (DMB-I + Placebo) - Group 2 (DMB-I) = 0.3963 Group 1 (DMB-I + Placebo) - Group 3 (Placebo) = 0.1616 Group 2 (DMB-I) - Group 3 (Placebo) = **0.0468**		

p-values indicating statistical significance of differences are shown in bold.

#### Dynamics of QOL-AD questionnaire assessment after 12 weeks of therapy (Visit 4) and after 26 weeks of therapy (Visit 6) compared with baseline (Visit 1)

Neither intra-group nor between-group analysis revealed statistically significant differences between groups in absolute values of assessment and changes in assessment on QOL-AD questionnaire.

#### Dynamics of assessment instrumental activities of daily living on IADL scale after 12 weeks of therapy (Visit 4) and after 26 weeks of therapy (Visit 6) compared with baseline level (Visit 1)

Significant improvement in dynamics of change from baseline in instrumental activity of daily living according to IADL scale was observed in group 2 (DMB-I, 60 mg/day) relative to group 3 (placebo) after 12 weeks of therapy (Visit 4) (*p* = 0.0472) (Table 5).

**Table 5:** Dynamics of changes in instrumental activities of daily living (IADL). FAS population.

Parameter/ Visit	Average score			Changes from baseline (Visit 1)		
	Group 1 (DMB-I + placebo)	Group 2 (DMB-I)	Group 3 (Placebo)	Group 1 (DMB-I + placebo)	Group 2 (DMB-I)	Group 3 (Placebo)
Visit 1						
N	47	41	45			
MEAN (SD)	4.49 (1.76)	4.51 (1.83)	4.64 (2.20)			
SEM	0.26	0.29	0.33			
Median; IQR	4.00; 3.00	4.00; 3.00	5.00; 4.00			
Q1; Q3	3.00; 6.00	3.00; 6.00	3.00; 7.00			
Min; Max	2.00; 8.00	2.00; 8.00	1.00; 8.00			
Range of variation	6.00	6.00	7.00			
p-value between groups (ANOVA/Kruskal-Wallis)	0.8250					
**Visit 4**						
N	46	40	44	46	40	44
MEAN (SD)	4.61 (1.78)	4.68 (1.80)	4.43 (2.00)	0.07 (0.53)	0.13 (0.65)	-0.16 (0.53)
SEM	0.26	0.29	0.30	0.08	0.10	0.08
Median; IQR	4.50; 3.00	4.00; 2.25	4.50; 3.00	0.00; 0.00	0.00; 0.00	0.00; 0.00
Q1; Q3	3.00; 6.00	3.75; 6.00	3.00; 6.00	0.00; 0.00	0.00; 0.00	0.00; 0.00
Min; Max	2.00; 8.00	2.00; 8.00	1.00; 7.00	-1.00; 2.00	-2.00; 2.00	-1.00; 1.00
Range of variation	6.00	6.00	6.00	3.00	4.00	2.00
p-value of change (paired T/U-test)				0.4370	0.2790	0.0572
p-value between groups (ANOVA/Kruskal-Wallis)	0.9330			**0.0366**		
p-value for pairwise comparisons (Tukey/Dunnett)				Group 1 (DMB-I + placebo) - Group 2 (DMB-I) = 0.5978 Group 1 (DMB-I + placebo) - Group 3 (Placebo) = 0.0748 Group 2 (DMB-I) - Group 3 (Placebo) = **0.0472**		
**Visit 6**						
N	44	40	44	44	40	44
MEAN (SD)	4.61 (1.63)	4.58 (1.81)	4.34 (2.02)	0.05 (0.68)	0.03 (0.86)	-0.25 (0.81)
SEM	0.25	0.29	0.30	0.10	0.14	0.12
Median; IQR	4.50; 3.00	4.00; 3.00	4.50; 3.00	0.00; 0.00	0.00; 0.00	0.00; 1.00
Q1; Q3	3.00; 6.00	3.00; 6.00	3.00; 6.00	0.00; 0.00	0.00; 0.00	-1.00; 0.00
Min; Max	2.00; 8.00	2.00; 8.00	1.00; 8.00	-1.00; 2.00	-3.00; 2.00	-2.00; 2.00
Range of variation	6.00	6.00	7.00	3.00	5.00	4.00
p-value of change (paired T/U-test)				0.6740	0.7630	0.0556
p-value between groups (ANOVA/Kruskal-Wallis)	0.8300			0.1190		

p-values indicating statistical significance of differences are shown in bold.

### Safety

A total of 145 AEs were identified in study, occurring in 61 patients (45.86% of SAF population). Majority — 135 AEs in 55 patients (41.35% of SAF population) — were mild. 17 AEs in form of dizziness, somnolence, lethargy, asthenia in 9 (6.77% of SAF population) patients were classified as expected (described in instructions for medicinal product Dimebon ). No severe or life-threatening AEs or deaths were recorded. When establishing a relationship with DMB-I intake, it was necessary to take into account nature of underlying disease, as well as concomitant cardiovascular pathology of patients in SAF sample. A presumptive association with DMB-I intake was noted for 31 AEs episodes in 18 (13.53% of SAF population) patients, most of which were mild in severity, regressed without additional therapy, and did not require discontinuation of study therapy.

## Discussion

In this study, we evaluate the efficacy of a DMB-I in combination with background therapy with memantine, which was administered at the same dose to all patients in all groups. Thus, the effect of background therapy was the same in all groups, and the effects of the study drug, the DMB-I, were evident during background therapy and could be objectively assessed. Memantine, an N-methyl-D-aspartate (NMDA) receptor antagonist, promotes glutamate regulation and has a positive effect on patients with moderate to severe AD, improving overall functioning, cognitive function, and daily activities^27^.

Our clinical trial met key requirements for clinical trials of new therapeutic agents for AD. First, we enrolled patients with a confirmed clinical diagnosis of mild to moderate dementia. Second, we used rating scales that are the gold standard for assessing the effectiveness of drugs aimed at improving cognitive function and overall well-being in patients with AD. Third, the study included preliminary statistical estimates of sample size, changes, and differences between groups^8^. ADAS-cog scale is mandatory in studies of effectiveness of drugs aimed at treating dementia in AD^37^. In our study, the primary efficacy measure was the difference in mean absolute changes from baseline in the ADAS-cog total score after 26 weeks of treatment between the DMB-I and placebo groups. Treatment success with the 60 mg/day DMB-I was determined by the difference in mean changes in ADAS-cog score compared with placebo, which was − 3.36 (-6.35, -0.37), and the change in score from baseline in the 60 mg/day DMB-I group, which was − 3.48 ± 5.23 points. The results of this primary efficacy measure obtained in our study are consistent with the results of other studies evaluating the efficacy of drugs for the treatment of dementia in AD. For example, according to a meta-analysis^38^, in donepezil versus placebo studies, changes in ADAS-cog scores in donepezil groups ranged from − 1.1 ± 5.36 to -4.32 ± 5.81, whereas changes in placebo groups ranged from + 0.58 ± 4.03 to -1.6 ± 5.69. The pooled mean difference and corresponding 95% confidence interval according to the meta-analysis results were − 0.28 (-0.39, -0.16)^39^. Similar results have been described for studies of other dementia drugs used as monotherapy (rivastigmine, galantamine, memantine)^38,39^. Combination therapy (ChEIs + memantine) moderately increases treatment efficacy^39,40^. 2024 meta-analysis^9^ compared intervention with placebo in terms of mean ADAS-cog scores from baseline to endpoint in patients with mild to moderate dementia associated with AD. In this meta-analysis, weighted means of this difference for two efficacy studies of 26-week treatment with memantine at a dose of 20 mg/day^41^ amounted to -1.23 points (95% CI -2.17 to -0.30). According to data of studies of galantamine therapy effectiveness, a meta-analysis established a difference with placebo for 32 mg/day of -3.29 points (95% CI -4.14 to -2.45), and for 24 mg/day − 3.03 points (95% CI -3.51 to -2.55)^42–44^. In donepezil studies, a meta-analysis found a difference of -1.95 points (95% CI -2.60 to -1.29) with 5 mg/day therapy and − 2.01 points (95% CI -2.64 to -1.39) with 10 mg/day therapy^45–49^. A meta-analysis of clinical trials of rivastigmine found a difference with placebo of -2.01 points (95% CI -2.70 to -1.32)^7,50^. Thus, efficacy for primary endpoint of efficacy assessment, namely difference from placebo in ADAS-cog scores from baseline to endpoint, in our study is comparable to registered and widely used drugs for treatment of dementia in AD.

An analysis of secondary efficacy endpoints in our study also indicates efficacy of DMB-I therapy. CGI-Efficacy index and CGI-I showed a decrease in total score, indicating improvement in patients’ overall condition after 3 months and after 6 months of DMB-I therapy at both doses. These results are comparable to those of donepezil studies^7,45^, memantine^41^ and galantamine^44^ where Clinician’s Interview-Based Impression of Change including caregiver information (CIBIC plus) scale variation was used; it also showed a decrease in scores.

In a 2024 meta-analysis (Chen et al., 2024), pooled incidence of AEs leading to patient discontinuation in groups receiving drugs versus placebo was 9.1% versus 4.5% for memantine 20 mg/day, 13.4% versus 6.7% for rivastigmine 12 mg/day, 26.8% versus 8.2% for galantamine 32 mg/day, 12.2% versus 6.9% for galantamine 24 mg/day, and 12.6% versus 6.9% for donepezil 10 mg/day. Moreover, a meta-analysis performed for four studies of 26-week therapy with Latrepirdine 60 mg/day^11,15,51,52^ shows that incidence of AEs leading to patient discontinuation was same for Latrepirdine and placebo - 6.3% versus 7.4%^31^. The same meta-analysis found an equal incidence of AEs for Latrepirdine and placebo: 60.4% versus 58.3%. Thus, existing safety data for six-month Latrepirdine therapy indicate a very favorable safety profile for this therapy, which was confirmed in our study, where overall incidence of AEs was 48.8% for 30 mg/day DMB-I dose, 41.5% for 60 mg/day DMB-I dose, and 45.9% for placebo.

Thus, DMB-I demonstrated a high safety profile in SAF population. In conclusion, clinical trial demonstrated potential of DMB-I for treatment of AD-associated dementia. The optimal dose, which demonstrated efficacy and safety, is 60 mg/day, and a Phase III trial is planned to obtain more extensive data. Phase III study will take place in 12 clinical sites in the Russian Federation and will begin by the end of year 2025^53^. Study will include 450 patients (175 patients will receive DMB-I at dose of 60 mg/day with memantine at dose of 20 mg/day; 175 patients will receive a placebo of DMB-I with memantine at dose of 20 mg/day; and 100 patients will receive DMB-I at dose of 60 mg/day as a monotherapy). Total duration of therapy with DMB-I will be increased to 52 weeks, that is in agreement with most part of guidelines of clinical investigation of medicines for treatment of AD^54,55^.

The main limitations of our study were that all participants were from the same country and had an unconfirmed diagnosis of AD. Patients did not undergo cerebrospinal fluid (CSF) analysis for validated CSF biomarkers (Aβ42, MTBR-tau243, other phosphorylated forms of tau) or positron emission tomography (PET) for amyloid or tau, which would have allowed for a definitive diagnosis^30^. CSF collection is an invasive and highly stressful procedure for elderly patients with dementia. The diagnosis of AD was presumptive and based on cognitive assessment and brain MRI analysis. However, all study participants had a definitive diagnosis of mild to moderate dementia using the MMSE-2 scale, the gold standard for assessing cognitive function in establishing a diagnosis of dementia^44,46^.

In addition, some exclusion criteria can be considered as study limitations, such as: cardiovascular disease (including a history of stroke), endocrine disorders such as diabetes, and moderate to severe depression. These conditions are common comorbidities in target group of elderly patients; furthermore, they are often associated with AD. The prevalence of comorbidities such as hypertension, diabetes (types 1 and 2), and stroke among patients with AD compared with controls has been assessed in studies conducted in United States and several countries of Europe and Asia^56^. Thus, hypertension in patients with AD is as common as in control patients^57^, diabetes, along with stroke^58^, is more common in patients with AD compared to control patients in most studies. Evidence from published studies indicates a multifactorial relationship between comorbidities such as cardiovascular disease and type 2 diabetes and the development of cognitive impairment^59,60^. In percentage terms, the prevalence of comorbidities among patients with AD ranges for hypertension from 30.2% in people with early-stage AD^61^ to 73.9% in users and non-users of acetylcholinesterase inhibitors, respectively^62^. Prevalence of diabetes ranges from 6.0% in people with AD dementia^63^ up to 24.3% (type 2 diabetes only)^64^. Prevalence of stroke ranges from 2.7% up to 13.7% (in people with late-onset AD)^61^. Regarding the exclusion of patients suffering from depression, this is a significant limitation, since depression is a condition that often accompanies AD and develops in parallel with the development of AD^65^. Thus, it was shown that in first year after diagnosis of AD, 51.4% of patients developed depression^66^. Conversely, a history of depression has also been shown to be associated with increased numbers of neuritic plaques and neurofibrillary tangles in hippocampus of patients with AD^67^. Despite of those limitations, applying exclusion criteria is a factor that ensures purity of experiment and facilitates detection and interpretation of both efficacy and side effects of test drug, since diseases included in the list of exclusion criteria may mask presence of an effect/side effect of study therapy.

## Conclusion

A 26-week treatment with DMB-I at a doses of 30 mg/day and 60 mg/day improved neurological outcomes in patients with AD compared to placebo, and DMB-I also demonstrated a good safety profile. Optimal dose of DMB-I was determined to be 60 mg/day, for which a long-term randomized controlled trial will be performed to confirm efficacy of DMB-I in everyday clinical practice.

## Data Availability

Data on the study protocol are available at [https://clinicaltrials.gov/study/NCT06292351](https:/clinicaltrials.gov/study/NCT06292351) (Accessed 07 January 2026). Detailed data from the study results are available upon request from corresponding author.

## List of abbrevations

FAS: Full analysis set
PP: Per protocol population
SAF: Safety population
AEs: Adverse events
SAEs: Serious adverse events
TEAE: Treatment-Emergent Adverse Even
SM: Study medication
MedDRA: Medical dictionary of terms of regulatory and legal activity
DMB-I: (Latrepirdine) polymorph
MMSE-2: Mini-Mental State Examination, 2ND Edition, www.parinc.com
ADAS-cog: Alzheimer’s Disease Assessment Scale–cognitive subscale
CGI: General clinical impression
CGI-S: Severity of the patient’s disorder
CGI-I: General improvement of condition
QOL-AD: Quality of life questionnaire in Alzheimer’s disease
IADL: Instrumental Activities of Daily Living
SD: Standard deviation
SEM: Standard error of mean
CI: Confidential interval
IQR: Interquartile range
Q3: 3rd quartile or 75% quantile
AD: Alzheimer’s disease
NMDA: N-methyl-D-aspartate
ChEIs: Cholinesterase inhibitors
Aβ: Beta amyloid
Tau: Tau-protein (neuron structural protein)
PET: Positron emission tomography
CSF: Cerebrospinal fluid
HIV: Human immunodeficiency virus
CT: Computed tomography
MRI: Magnetic resonance imaging
eCRF: electronic case report form
FDA: Food and Drug Administration
